# Apoptotic bodies derived from mesenchymal stem cells promote cutaneous wound healing via regulating the functions of macrophages

**DOI:** 10.1186/s13287-020-02014-w

**Published:** 2020-11-27

**Authors:** Jin Liu, Xinyu Qiu, Yajie Lv, Chenxi Zheng, Yan Dong, Geng Dou, Bin Zhu, Anqi Liu, Wei Wang, Jun Zhou, Siying Liu, Shiyu Liu, Bo Gao, Yan Jin

**Affiliations:** 1 State Key Laboratory of Military Stomatology& National Clinical Research Center for Oral Diseases & Shaanxi International Joint Research Center for Oral Diseases, Center for Tissue Engineering, School of Stomatology, The Fourth Military Medical University, Xi’an, 710032 Shaanxi China; 2 State Key Laboratory of Military Stomatology & National Clinical Research Center for Oral Diseases & Shaanxi Key Laboratory of Stomatology, Department of Prosthodontics, School of Stomatology, The Fourth Military Medical University, Xi’an, 710032 Shaanxi China; 3 Department of Dermatology, Tangdu Hospital, The Fourth Military Medical University, Xi’an, 710038 China; 4 Department of Stomatology, General Hospital of Tibet Military Region, Lhasa, 850007 Tibet China; 5 State Key Laboratory of Military Stomatology, National Clinical Research Center for Oral Diseases & Shaanxi Key Laboratory of Oral Diseases, Department of Operative Dentistry and Endodontics, Fourth Military Medical University, Xi’an, China

**Keywords:** Apoptotic bodies, Extracellular vesicles, Macrophage polarization, Skin regeneration

## Abstract

**Background:**

As the major interface between the body and the external environment, the skin is liable to various injuries. Skin injuries often lead to severe disability, and the exploration of promising therapeutic strategies is of great importance. Exogenous mesenchymal stem cell (MSC)-based therapy is a potential strategy due to the apparent therapeutic effects, while the underlying mechanism is still elusive. Interestingly, we observed the extensive apoptosis of exogenous bone marrow mesenchymal stem cells (BMMSCs) in a short time after transplantation in mouse skin wound healing models. Considering the roles of extracellular vesicles (EVs) in intercellular communication, we hypothesized that the numerous apoptotic bodies (ABs) released during apoptosis may partially contribute to the therapeutic effects.

**Methods:**

ABs derived from MSCs were extracted, characterized, and applied in mouse skin wound healing models, and the therapeutic effects were evaluated. Then, the target cells of ABs were explored, and the effects of ABs on macrophages were investigated in vitro.

**Results:**

We found ABs derived from MSCs promoted cutaneous wound healing via triggering the polarization of macrophages towards M2 phenotype. In addition, the functional converted macrophages further enhanced the migration and proliferation abilities of fibroblasts, which together facilitated the wound healing process.

**Conclusions:**

Collectively, our study demonstrated that transplanted MSCs promoted cutaneous wound healing partially through releasing apoptotic bodies which could convert the macrophages towards an anti-inflammatory phenotype that plays a crucial role in the tissue repair process.

**Supplementary Information:**

The online version contains supplementary material available at 10.1186/s13287-020-02014-w.

## Introduction

The skin is the largest organ of the human body which serves as the first defense line to the external environment, so that it is liable to different kinds of injuries. Large area loss of the skin often leads to severe disability or even death, which not only physically and mentally suffers the patients, but also brings a heavy burden to the society [1]. To date, various strategies have been explored, among which exogenous mesenchymal stem cells (MSCs) showed a great potential [2]. More and more studies have demonstrated that MSCs could accelerate cutaneous wound healing and restore the more integrate and organized skin structure [3–5]. However, the underlying mechanism remains elusive. Nowadays, paracrine pathway is increasingly approved, by which MSCs release growth factors, cytokines, and extracellular vesicles beneficial to the wound healing process [6–8]. Interestingly, we previously observed extensive apoptosis of transplanted MSCs in a short time after transplantation in a rabbit skin wound healing model and found that apoptosis has an important role in the activation of the inflammatory regulatory abilities of MSCs [9]. Likewise, using a murine model of graft-versus-host disease (GvHD), another study demonstrated that MSCs undergo extensive caspase activation and apoptosis after transplantation, which is necessary for their immunosuppressive function [10].

Apoptosis refers to the programmed cell death, which is of great importance in the development, growth, and immune processes of organisms [11]. During apoptosis, cells undergo a series of morphological changes and eventually form apoptotic bodies (ABs) [12]. ABs are the largest kind of extracellular vesicles with a rich cargo like DNA, RNA, proteins, and organelles [13]. As it contains multiple contents, we supposed that ABs could contribute to the therapeutic effects of MSCs after transplantation. Moreover, once apoptosis occurs, apoptotic signals are transmitted promptly to phagocytes, so that apoptotic cells and debris can be cleared in a short time [14]. Macrophages are the professional phagocytes which are responsible for the clearance of apoptotic issues [15]. Meanwhile, macrophages are important immune cells, which play essential roles both in inflammation and tissue repair processes [16]. Thereby, we inferred that macrophages are the target cells of ABs.

In this study, we verified the therapeutic effects of transplanted MSCs in mouse skin wound healing models and observed vast apoptosis of transplanted MSCs in a short time. Then, we extracted ABs and utilized them to demonstrate that ABs could accelerate the cutaneous wound healing process. Meanwhile, we found that macrophages were probably the target cells of ABs both in vivo and in vitro experiments and proved that ABs could promote macrophage polarization towards the M2 phenotype. Finally, M2 phenotypic macrophages further enhanced the function of fibroblasts, which synergistically promoted cutaneous wound healing. In conclusion, we revealed the underlying mechanism for exogenous MSC-based therapy and provided a new therapeutic strategy for skin injuries.

## Materials and methods

### Animals

All procedures were approved by the Institutional Animal Care and Use Committee of the Fourth Military Medical University and conformed to the Guide for the Care and Use of Laboratory Animals published by the National Institutes of Health. All animals were purchased from the Laboratory Animal Center of the Fourth Military Medical University. Eight-week-old female wild type (WT) C57BL/6 mice were used both for isolation of cells and establishment of skin wound healing models. The mice were kept under specific pathogen-free conditions (24 °C, 12-h light/dark cycles, and 50% humidity) with free access to food and water.

### Cell culture

Isolation and culture of murine bone marrow mesenchymal stem cells (BMMSCs) were performed as previously described [17]. Eight-week-old female WT C57BL/6 mice (18–20 g) were used. The hindlimbs were removed, and then bone marrow cells were separated by flushing marrow cavities and seeded in a medium of alpha-minimum essential medium (α-MEM) (Gibco, USA) supplemented with 20% fetal bovine serum (FBS) (Gibco, USA), 2 mM l-glutamine, 100 U/ml penicillin, and 100 g/ml streptomycin (all from Invitrogen, USA). For in vivo tracing, BMMSCs were pre-labeled with PKH26 (Sigma-Aldrich, USA) according to the manufacturers’ protocol and detected by the confocal microscope (Nikon, Japan).

Bone marrow-derived monocytes (BMDMs) were also isolated from hindlimbs. Bone marrow cells were separated and washed in Red Blood Cell Lysis Buffer (Beyotime, China). The cell suspension was centrifuged at 100*g* for 5 min and then the cell pellet was resuspended and seeded in a medium of high-glucose Dulbecco’s modified Eagle medium (DMEM) (Gibco, USA) supplemented with 10% FBS, 2 mM l-glutamine, 100 U/ml penicillin, and 100 g/ml streptomycin. Cells were cultured for 7 days in the presence of recombinant mouse macrophage colony-stimulating factor (M-CSF) (R&D, USA) at 20 ng/ml to induce mature macrophages. After 7 days, macrophages were added with lipopolysaccharide (LPS) (Sigma-Aldrich, USA) at 1 μg/ml for 24 h to stimulate inflammation.

Primary fibroblasts were isolated from neonatal murine skin tissue. After enzymatic digestion in dispase (Gibco, USA) overnight at 4 °C, the dermis was separated from the epidermis. Then, the dermis was minced into small pieces and incubated in type I collagenase (Gibco, USA) for 1 h at 37 °C with manual agitation every 15 min. The cell suspension was filtrated with a 100-mesh sieve and centrifuged at 100*g* for 5 min. The cell pellet was resuspended and seeded in a medium of α-MEM supplemented with 10% FBS, 2 mM l-glutamine, 100 U/ml penicillin, and 100 g/ml streptomycin.

All cells were cultured at 37 °C in a humidified atmosphere of 5% CO_2_. Cells were digested and passaged by using 0.25% trypsin (Invitrogen, USA) at 80–90% confluence.

### Isolation and characterization of apoptotic bodies

Apoptotic bodies (ABs) were isolated by using the optimized protocol. Firstly, BMMSCs were treated with staurosporine (STS) (Cell Signaling Technology, USA) at 0.5 μM for 12 h to induce apoptosis. The supernatant was collected and centrifuged at 1000*g* for 10 min at 4 °C to remove cells and debris. Then, the supernatant was further centrifuged at 16000*g* for 30 min at 4 °C, and the pellet was washed twice in phosphate-buffered saline (PBS). The isolated ABs were suspended in 100 μl PBS and stored at − 80 °C for further study if necessary. ABs were quantified by BCA assay (TIANGEN, China) before using. The morphological features of ABs were observed by a scanning electron microscopy (SEM) (Hitachi, Japan), and the size distribution was measured by dynamic light scattering (DLS) analysis by using Zetasizer Nano ZSE (Malvern, UK). The expression of C1q and Annexin V in ABs were examined by immunofluorescent staining. The Annexin V staining was carried out by using an Annexin V-FITC apoptosis assay kit (7Sea Biotech, China). The protein expression levels of caspase-3 in BMMSCs and ABs were detected by western blots. To detect phagocytosis both in vivo and in vitro, ABs were labeled with PKH26 and detected by a confocal microscope. The primary antibodies involved in this study include C1q (1:200) (CEDARLANE, CL7501F, monoclonal, Canada), β-actin (1:1000) (CWBio, CW0096, monoclonal, China), and caspase-3 (1:1000) (Cell Signaling Technology, 9662, polyclonal, USA).

### Flow cytometric analysis of cell phenotype

For flow cytometric analysis of the cell surface markers, MSCs were detached by using 0.25% trypsin and resuspended in PBS supplemented with 3% FBS. Then, the cells were incubated in dark at 4 °C for 30 min with phycoerythrin (PE)-conjugated mouse anti-CD29 (46-0299-41, monoclonal), anti-CD34 (15-0349-41, monoclonal), anti-CD105 (12-1057-42, monoclonal), anti-CD146 (12-1469-42, monoclonal) and fluorescein isothiocyanate (FITC)-conjugated mouse anti-CD11b (11-0112-82, monoclonal), anti-CD45 (11-0451-82, monoclonal), anti-CD90 (11-0900-81, monoclonal), and anti-Sca-1 (11-5981-82, monoclonal) (all from eBioscience, USA), respectively. Related conjugated IgG (eBioscience, USA) was used as the negative control. Finally, cells were washed twice in PBS and positively stained cells were detected by the flow cytometer (Beckman Coulter, USA).

### Osteogenic differentiation assay

MSCs were seeded in six-well plates at a density of 5 × 10^5^ cells per well. When cells reached 100% confluence, the basal medium was changed into osteogenic induction medium: α-MEM containing 10% FBS, 1% penicillin/streptomycin, 5 mM β-glycerophosphate, 50 μg/ml ascorbic acid, and 10 nM dexamethasone (all from Sigma-Aldrich, USA). The medium was refreshed every other day. After 7 days of osteogenic induction, alkaline phosphatase (ALP) staining was performed with the commercial kit (Beyotime, China). After 21 days of osteogenic induction, Alizarin red staining was performed. Photographs were taken by using an inverted optical microscope (Olympus, Japan).

### Adipogenic differentiation assay

MSCs were seeded in six-well plates at a density of 5 × 10^5^ cells per well. When cells reached 100% confluence, the basal medium was changed into adipogenic induction medium: α-MEM containing 10% FBS, 0.5 mM isobutylmethylxanthine, 0.5 mM dexamethasone, and 60 nM indomethacin (all from Sigma-Aldrich, USA). The medium was refreshed every other day. After 7 days of adipogenic induction, Oil Red O staining was performed to indicate lipid droplet formation. Photographs were taken by using an inverted optical microscope (Olympus, Japan).

### Colony-forming unit assays

MSCs were seeded in 5-cm culture dishes at a density of 1 × 10^4^ cells per dish and cultured in basal medium for 2 weeks. The medium was refreshed every 3 days until colonies with over 50 cells were observed. The colonies were stained with 0.2% crystal violet (Sigma-Aldrich, USA), and photographs were taken by using an inverted optical microscope (Olympus, Japan).

### Skin wound healing models

Eight-week-old female WT C57BL/6 mice (18–20 g) were used. Thirty-six mice underwent anesthesia by an intraperitoneal injection of pentobarbitone sodium (40 mg/kg). After shaving and cleaning, a full-thickness wound (1 cm in diameter) was created on the dorsal skin. The mice were randomly divided into four groups: PBS group, Gel group, Gel+MSCs group, and Gel+ABs group. Commercial hydrogel Pluronic F-127 (PF-127) (Sigma-Aldrich, USA) was dissolved in PBS at a concentration of 30% at 4 °C. MSCs and ABs were embedded in PF-127 solution at 4 °C. The number of MSCs was 2 × 10^6^ (in 100 μl PF-127 solution) when they were applied to the wound site, which was calculated by the digital cell counter (Bio-Rad, USA). The amount of ABs was 50 μg (in 100 μl PF-127 solution) when they were applied to the wound site, which was measured by BCA assay (TIANGEN, PA115). The treatments were conducted on days 0, 3, and 7, and the dressings were kept on the wound site until the next time point. After the operation, the wound was covered by surgical dressings (3 M, USA) and the mice were housed individually. The wound area and body weight were measured on days 0, 3, 7, 10, and 14 post-surgery, and the wound healing rate was calculated according to the formula: D_*n*_ wound healing rate = (D_0_ wound area − D_*n*_ wound area)/D_0_ wound area × 100%. The mice were sacrificed on day 14, and the skin tissues were harvested for further analysis.

### Immunofluorescent staining

Cells and skin tissue samples were fixed in 4% paraformaldehyde overnight. Skin tissue samples underwent dehydration with 30% saccharose and were embedded in optimal cutting temperature compound (OCT) (Leica, Germany) and cut into 10-μm-thick sections. The cells and sections underwent permeabilization with 0.05% Triton X-100 (Sigma-Aldrich, USA) for 10 min at room temperature, blockage with 5% bovine serum albumin (BSA) (Sigma-Aldrich, USA) at 37 °C for 30 min, and incubation with the primary antibody overnight at 4 °C. Next, the sections were incubated with fluorescence secondary antibody (Sigma-Aldrich, USA) at room temperature for 1 h. Lastly, the nuclei were counterstained by Hoechst 33342 (Sigma-Aldrich, USA) for 10 min at room temperature. Photographs were taken by a confocal microscope and evaluated by ImageJ software. The primary antibodies involved in this study include F4/80 (1:200, ab6640, monoclonal), Cytokeratin 14 (1:400, ab181595, monoclonal), CD206 (1:200, ab195191, monoclonal), and Ki67 (1:150, ab16667, monoclonal) (all from Abcam, UK).

### Histological staining

Skin tissue samples were fixed in 4% paraformaldehyde for 24 h and underwent dehydration with graded ethanol. Then, the samples were embedded in paraffin and cut into 4-μm-thick sections. H&E staining and Masson staining were carried out by using the commercial staining kits (Baso technology, China).

### Cell migration assay

Fibroblasts were seeded in 6-well plates at a density of 5 × 10^5^ cells per well. When cells reached 100% confluence, the scratch was made on the plates by using sterile pipette tips. Cells were cultured in medium cocultured with ABs at 5 μg/ml, 10 μg/ml, 20 μg/ml, and 30 μg/ml respectively, or conditioned medium collected from BMDM. Cells cultured in serum-free medium were used as the negative control. Photographs were taken by an inverted microscope at 0 h, 12 h, and 24 h and evaluated by ImageJ software.

### RNA extraction and qPCR

BMDMs were treated with ABs at 5 μg/ml, 10 μg/ml, 20 μg/ml, and 30 μg/ml respectively. The culture medium was discarded, and the total RNA of BMDMs was extracted by using Trizol Reagent (Takara, Japan) 24 h later. After that, complementary DNA (cDNA) was synthesized using a PrimeScript RT reagent kit (Takara, Japan). Then, qPCR procedure was performed with CFX96TM system (Bio-Rad, USA) using a SYBR Premix Ex Taq II kit (Takara, Japan). *Gapdh* was used as the internal control. All the primer sequences are listed in Table 1.

**Table 1:** Primer sequences

Gene	Forward	Reverse
*Gapdh*	5′-TGTGTCCGTCGTGGATCTGA-3′	5′-TTGCTGTTGAAGTCGCAGGAG-3′
*Il-10*	5′-GCCAGAGCCACATGCTCCTA-3′	5′-GATAAGGCTTGGCAACCCAAGTAA-3′
*Arg-1*	5′-AGCTCTGGGAATCTGCATGG-3′	5′-ATGTACACGATGTCTTTGGCAGATA-3′
*Tgf-β*	5′-CAAGCTGAACTTGAGCGAGGA-3′	5′-TTTACTCAGTGCCAGAAGCTGGA-3′

### ELISA

Culture medium from BMDMs was collected after treatment with ABs at 5 μg/ml, 10 μg/ml, 20 μg/ml, and 30 μg/ml respectively. To exclude the production of TGF-β and/or IL-10 by ABs themselves, we added ABs (30 μg/ml) into the culture medium (without BMDMs) and put the medium in a humidified atmosphere of 5% CO_2_. Then, the medium was collected 24 h later. The concentrations of IL-10 and TGF-β in the conditioned medium were measured by using murine ELISA kits (NeoBioscience, China) according to the protocols described by the manufacturers.

### TUNEL staining

TdT-mediated dUTP nick-end labeling (TUNEL) staining was performed as manufacturers’ protocols with the in situ cell death detection kit in frozen sections (Roche, Switzerland). Briefly, approximately 10-μm-thick sections were prepared from the skin tissues. After sequential pretreatment, each section was stained with TUNEL reagents for 1.5 h at 37 °C in the dark environment and Hoechst 33342 was used to counterstain the nuclei. Digital photographs were taken by a confocal microscope (Olympus, Japan).

### Western blots

BMDMs were treated with ABs at 5 μg/ml, 10 μg/ml, 20 μg/ml, and 30 μg/ml respectively. The culture medium was discarded, and the proteins from BMDMs were extracted by using RIPA buffer with protease inhibitor (Beyotime, China) 24 h later. After being quantified by BCA assay, the proteins were loaded on sodium dodecyl sulfate-polyacrylamide (SDS) gels and transferred to polyvinylidene fluoride (PVDF) membranes (Millipore, USA). Then, the membranes were blocked with 5% BSA for 2 h at room temperature and incubated with primary antibodies overnight at 4 °C. Finally, the membranes were incubated with peroxidase-conjugated secondary antibodies (CWBio, China) for 1 h at room temperature. The protein bands were detected by the imaging system (Tanon, China) and quantified by ImageJ software. Primary antibodies include GAPDH (1:1000) (CWBio, CW0100, monoclonal, China), ARG-1 (1:400) (Cell Signaling Technology, 93668, monoclonal, USA), and TGF-β (1:500) (Abcam, ab215715, monoclonal, UK).

### Statistical analysis

All the data was expressed as the mean ± standard deviation (SD). Comparisons of two groups were performed by two-tailed Student’s *t* tests, and comparisons of multiple groups were performed by one-way analysis of variance (ANOVA) with *Tukey* correction by using the Statistical Program for Social Science software (IBM, Armonk, NY, USA). *P* < 0.05 was considered as statistically significant. Data tables are presented in additional files summarizing the mean and standard deviation of the data obtained (Additional Tables 1–7).

## Results

### Transplanted MSCs promoted cutaneous wound healing after transplantation

**Fig. 1:**
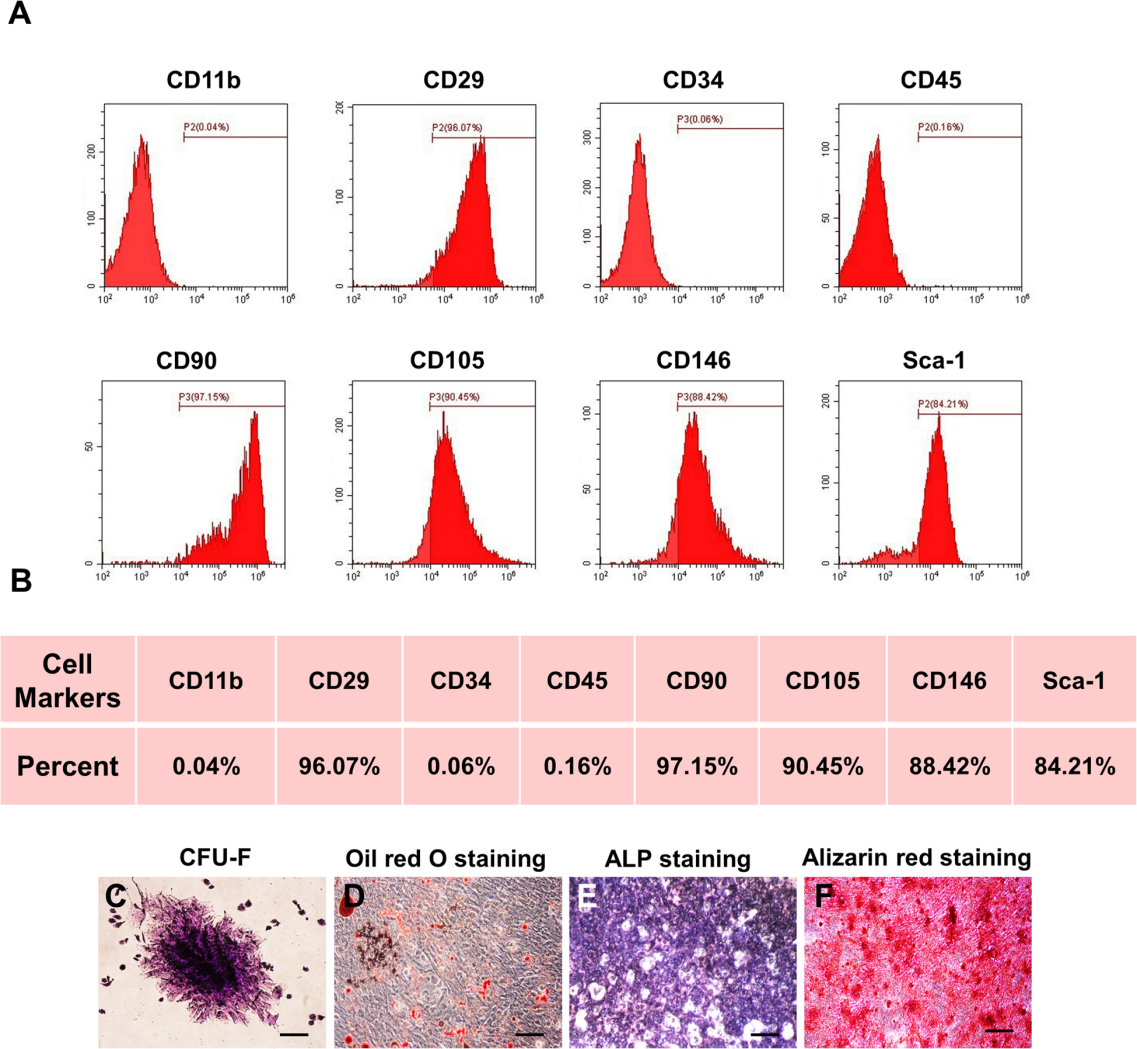
Characterization of murine BMMSCs. **a**, **b** Flow cytometric analysis of the surface markers showed positive expression of CD29, CD90, CD105, CD146, and Sca-1, and negative expression of hematopoietic markers CD11b, CD34, and CD45. **c** Representative image of colony formation of murine BMMSCs. **d** Representative image of Oil Red O staining of murine BMMSCs. **e** Representative image of ALP staining of murine BMMSCs. **f** Representative image of Alizarin red staining of murine BMMSCs. Scale bar, 500 μm

**Fig. 2:**
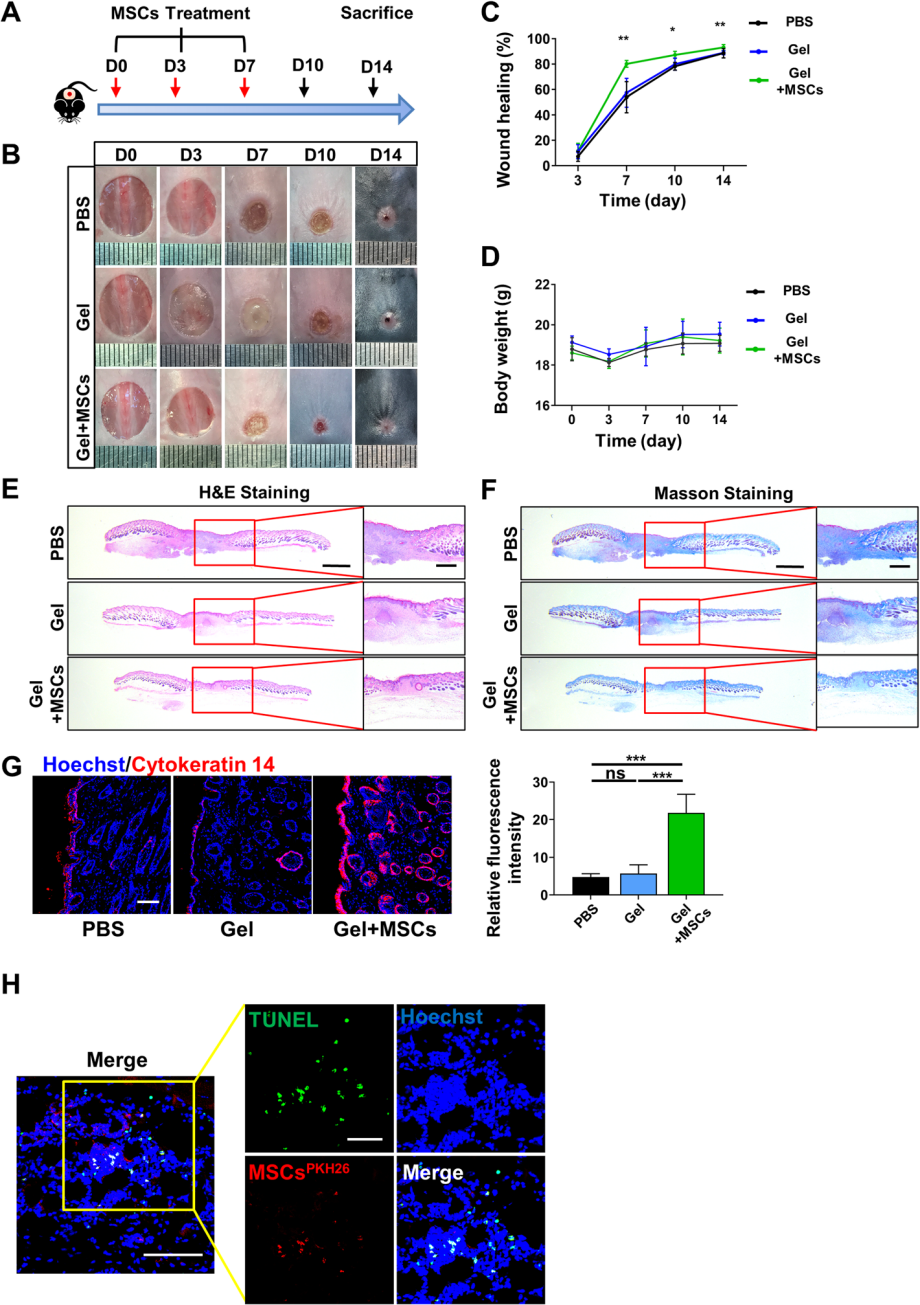
Transplanted MSCs promoted cutaneous wound healing while underwent apoptosis. **a** The schematic graph shows the protocol of establishment and treatment for mouse skin wound models. **b** Representative photographs of cutaneous wounds in different groups at different time points during the wound healing procedure. **c** Quantification of the wound healing rate. **d** Quantification of the body weight. **e** Representative images of the H&E staining of the skin samples. Scale bar, 1 mm in low-magnification images, 500 μm in high-magnification images. **f** Representative images of the Masson staining of the skin samples. Scale bar, 1 mm in low-magnification images, 500 μm in high-magnification images. **g** Representative images and quantification of the cytokeratin 14 expression in the skin samples. Scale bar, 100 μm. **h** Representative images of TUNEL staining of skin tissue. Scale bar, 100 μm in low-magnification images, 50 μm in high-magnification images. *n* = 6 per group. Data shown as mean ± SD. *P < 0.05; **P < 0.01; ***P < 0.001; NS, not significant. PBS, phosphate-buffered saline; Gel, PF-127 gel; Gel+MSCs, MSCs embedded in PF-127; MSCs^PKH26^, MSCs labeled with PKH26

Bone marrow mesenchymal stem cells (BMMSCs) were isolated from 8-week-old female WT C57BL/6 mice, and the second passage was used for characterization. We firstly analyzed the expression of surface markers by flow cytometry. The MSCs we isolated highly expressed CD29, CD90, CD105, CD146, and Sca-1, while hematopoietic markers like CD11b, CD34, and CD45 were not detected (Fig. 1a, b). Then, we examined the colony formation property by using colony-forming unit assays (Fig. 1c). Meanwhile, we detected the osteogenic and adipogenic differentiation property by ALP staining, Alizarin red staining, and Oil Red O staining, respectively (Fig. 1d–f). In order to verify the therapeutic efficacy of the transplanted MSCs, we established the mouse skin wound healing models and locally employed exogenous MSCs for treatment (Fig. 2a). MSCs were embedded in PF-127, a biocompatible, nontoxic, and thermo-reversible material, which has been widely applied in many studies [18, 19]. With a proper concentration, PF-127 solution is a free-flowing liquid at room temperature, but forms a hydrogel at body temperature, which can stay safely on the wound area [20]. Meanwhile, PBS was used as a blank control and PF-127 was used alone to exclude the effect of the gel. Photographs of the wound area were taken at five different time points during the wound healing procedure, and the wound healing rate was calculated as well (Fig. 2b, c). As we expected, the group treated with transplanted MSCs healed the fastest among the three groups, while no significant differences were found in the wound healing rate of the other two groups (Fig. 2c). We then collected the skin samples at day 14 and further conducted a histological analysis to assess the result of skin regeneration. As shown by H&E and Masson staining (Fig. 2e, f, Additional file 1A-B), MSCs significantly enhanced the effect on cutaneous wound healing compared to that observed in the PBS or Gel groups. In the MSC group, we observed smaller scar areas, less infiltration of inflammatory cells, and a more integrate cutaneous structure with newly formed epithelium, some hair follicles as well as better deposited and organized collagen. In addition, we detected the expression level of cytokeratin 14 by immunofluorescent staining (Fig. 2g). Cytokeratin 14 is a marker of keratinocytes, the levels of which present the quality of re-epithelization in cutaneous wound healing [21]. The fluorescence intensity was significantly higher in the MSC group, which implied better re-epithelization in this group than the other two groups. All these data demonstrated that the transplanted MSCs accelerated the cutaneous wound healing rate and enhanced the cutaneous regeneration. To explore apoptosis of transplanted MSCs in vivo, we labeled the cells with PKH26 and detected apoptosis by TdT-mediated dUTP nick-end labeling (TUNEL) staining 24 h later after transplantation (Fig. 2h). Numerous PKH26-labeled MSCs were also TUNEL positive, which means the transplanted MSCs underwent apoptosis after transplantation. In summary, the transplanted MSCs promoted cutaneous wound healing while underwent apoptosis in a short time.

### MSC-derived ABs promoted cutaneous wound healing

**Fig. 3:**
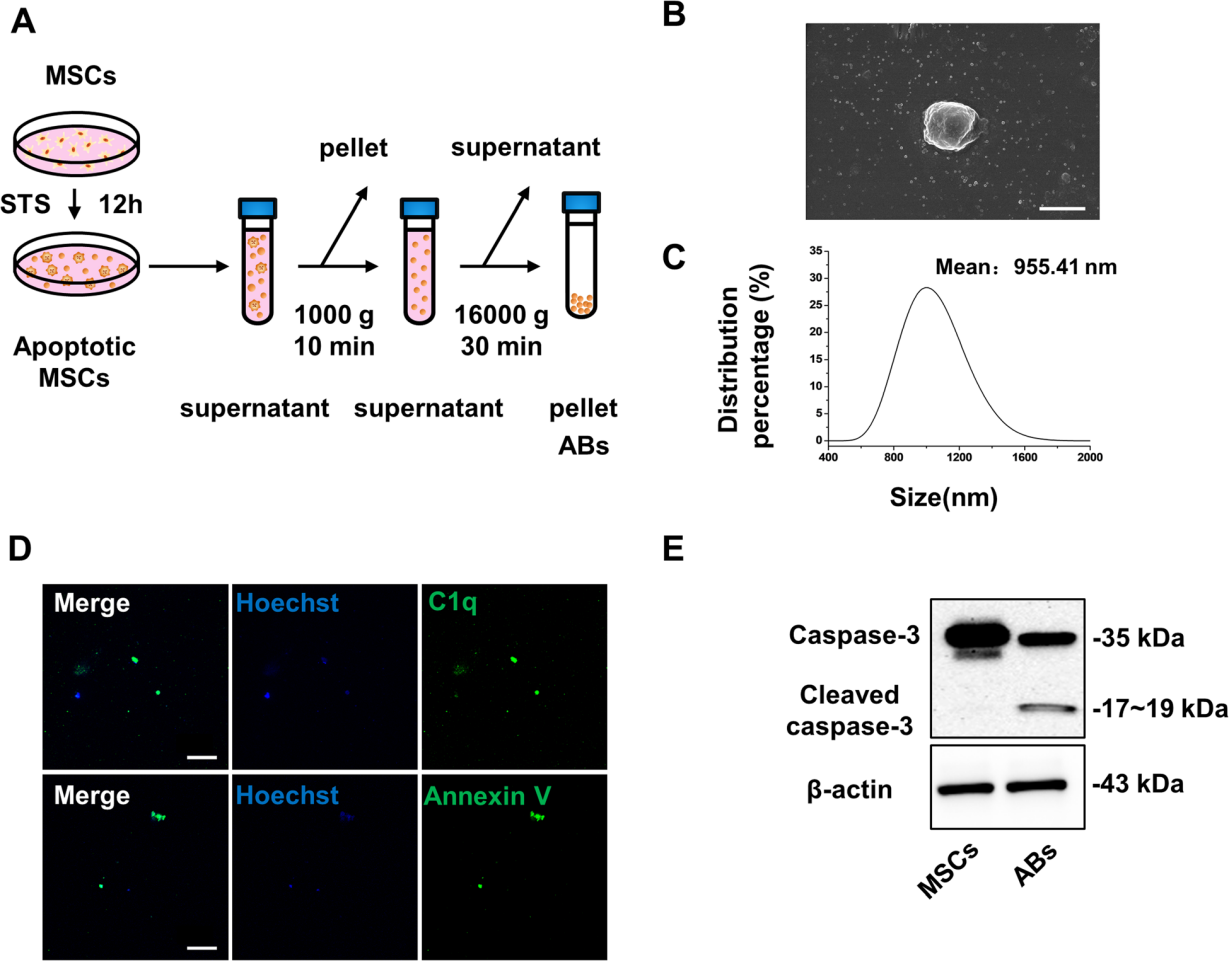
Characterization of apoptotic bodies from MSCs (MSC-ABs). **a** The schematic graph shows the protocol of isolation of MSC-ABs. **b** Representative image of SEM analysis of MSC-ABs. Scale bar, 1 μm. **c** Size distribution of MSC-ABs measured by DLS. **d** Representative images of C1q and Annexin V staining of MSC-ABs. Scale bar, 20 μm. **e** The protein level of caspase-3 for MSC-ABs examined by western blots

ABs were isolated by using the optimized gradient centrifugation protocol and then underwent characterization (Fig. 3a). Firstly, we observed the morphology and size distribution of ABs by SEM and dynamic light scattering (DLS) analysis (Fig. 3b, c). Then, we detected the expression of C1q and Annexin V in ABs by immunofluorescent staining (Fig. 3d). In addition, protein expression levels of caspase-3 and cleaved caspase-3 were detected by western blots (Fig. 3e). The results showed the ABs we used in this study were vesicles as approximately 1 μm in size, with C1q and Annexin V positive and cleaved caspase 3 expressed.

**Fig. 4:**
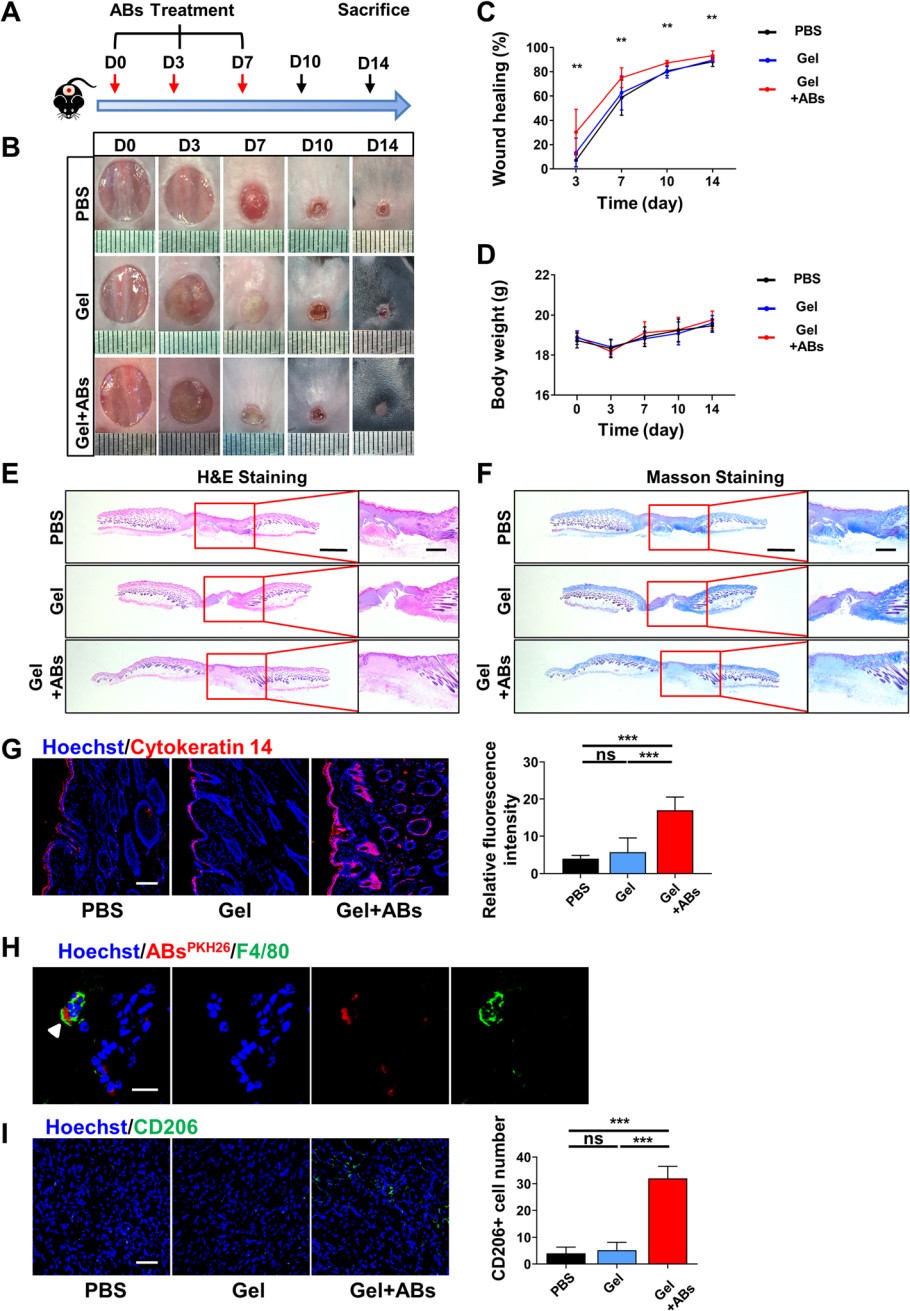
ABs derived from MSCs promoted cutaneous wound healing. **a** The schematic graph shows the protocol of establishment and treatment for mouse skin wound models. **b** Representative photographs of cutaneous wounds in different groups at different time points during the wound healing procedure. **c** Quantification of the wound healing rate. **d** Quantification of the body weight. **e** Representative images of the H&E staining of the skin samples. Scale bar,1 mm in low-magnification images, 500 μm in high-magnification images. **f** Representative images of the Masson staining of the skin samples. Scale bar, 1 mm in low-magnification images, 500 μm in high-magnification images. **g** Representative images and quantification of the cytokeratin 14 expression in the skin tissue. Scale bar, 100 μm. **h** Immunofluorescence staining shows ABs engulfed by F4/80-positive macrophages in vivo. Scale bar, 20 μm. **i** Representative images and quantification of the CD206 expression in the skin tissue. Scale bar, 50 μm. *n* = 6 per group. Data shown as mean ± SD. *P < 0.05; **P < 0.01; ***P < 0.001; NS, not significant. PBS, phosphate-buffered saline; Gel, PF-127 gel; Gel+ABs, ABs embedded in PF-127 gel; ABs^PKH26^, ABs labeled with PKH26

To investigate the therapeutic efficacy of ABs, we locally administrated ABs in mouse skin wound healing models by using the same methods with MSC-based therapy (Fig. 4a). The group treated with ABs healed the fastest among the three groups, while no significant differences were found in the wound healing rate compared with the other two groups (Fig. 4b, c). H&E and Masson staining showed that ABs significantly enhanced cutaneous wound healing compared to that observed in the PBS or Gel groups. In the AB-loaded group, we observed smaller scar areas, less infiltration of inflammatory cells, and a more integrate cutaneous structure with newly formed epithelium, some hair follicles as well as better deposited and organized collagen (Fig. 4e–f, Additional file 2A-B). Immunofluorescent staining for cytokeratin14 showed the higher fluorescence intensity in the AB-loaded group (Fig. 4g). These data demonstrated that using ABs alone accelerated the cutaneous wound healing rate and enhanced the quality of skin regeneration. To confirm the fate of ABs, we labeled ABs with PKH26 and traced them in vivo. We used F4/80 to mark macrophages, which is expressed on the cell membrane. As shown in Fig. 4h, the cells indicated by the arrow are F4/80 positive, which should be macrophages. ABs labeled with PKH26 are surrounded by the macrophages. Therefore, we estimated that ABs are probably engulfed by F4/80-positive macrophages 6 h after treatment. Since F4/80-positive macrophages were probably the target cells of ABs, we next tested the effects of ABs on macrophages. Since M2 phenotypic macrophages are anti-inflammatory and play critical roles in tissue repair, we detected the expression levels of CD206, a marker of M2 phenotypic macrophages, by immunofluorescent staining (Fig. 4i). It is worth noting that CD206-positive cell numbers were much more in the AB-loaded group than in the other two groups. The results showed that the group treated with ABs significantly expressed more CD206 than the other two groups 14 days after surgery. However, CD206 is also expressed in immature dendritic cells, which made it difficult to specify the changes of macrophages. To determine whether ABs has a direct effect on macrophage polarization, we next carried out several relevant experiments in vitro.

### MSC-derived ABs promoted polarization of macrophages towards M2 phenotype

**Fig. 5:**
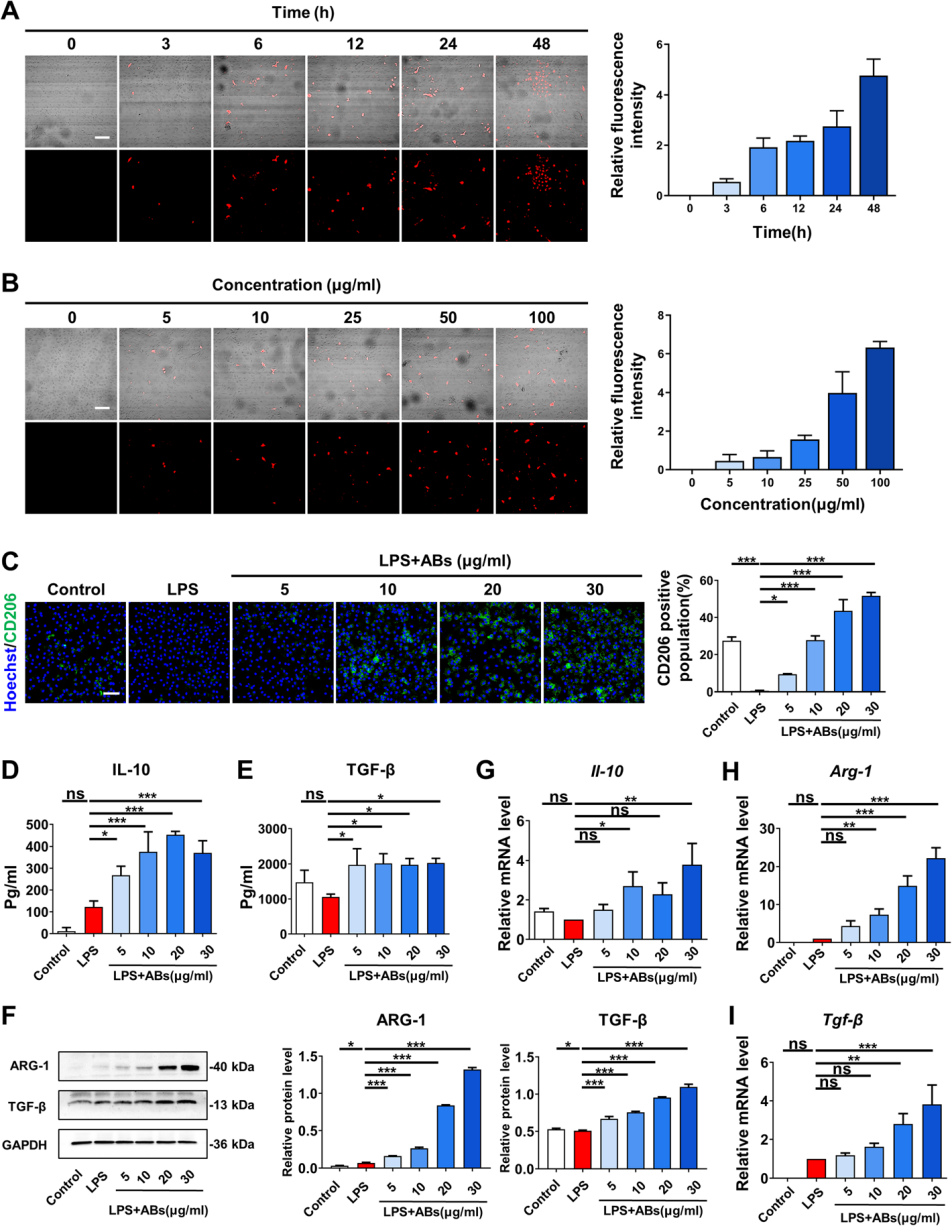
ABs promoted macrophage polarization towards the M2 phenotype. **a** The immunofluorescence images of time-dependent uptake of ABs by macrophages. Scale bar, 100 μm. **b** The immunofluorescence images of concentration-dependent uptake of ABs by macrophages. Scale bar, 100 μm. **c** The immunofluorescence images and quantification of the CD206 expression level of macrophages treated with different concentrations of ABs. Scale bar, 100 μm. **d**, **e** The detection of secretory factors IL-10 (**d**) and TGF-β (**e**) released by macrophages treated with different concentrations of ABs by ELISA. **f** The protein levels of ARG-1 and TGF-β of macrophages treated with different concentrations of ABs examined by western blots. **g**–**i** The gene expression level of *Il-10* (**g**), *Arg-1* (**h**), and *Tgf-β* (**i**) of macrophages treated with different concentrations of ABs examined by qPCR. *n* = 3 per group. Data shown as mean ± SD. *P < 0.05; **P < 0.01; ***P < 0.001; NS, not significant

Since we observed that macrophages were probably the target cells of ABs in vivo and an increase number of CD206-positive cells, we further explored the effects of ABs on macrophage polarization in vitro. Macrophages are highly heterogeneous cells which present distinct properties fitting different organs and tissues [22]. In healthy skin, it is resident macrophages originating from the embryo that involve in homeostasis maintaining, while in damaged skin, circulating monocytes from the bone marrow are recruited into injury sites and differentiate into macrophages to aid clearance of damaged cells [23]. Firstly, we labeled ABs with PKH26 and then examined the phagocytosis of macrophages (Fig. 5a, b). The results showed that macrophages engulfed ABs in a time- and concentration-dependent way. Then, we used lipopolysaccharide (LPS) as a simple in vitro technically to mimic the inflammatory microenvironment for macrophages [16]. After stimulated by LPS, macrophages were treated with different concentrations of ABs and the unstimulated macrophages were used as control. We detected the expression levels of CD206 by immunofluorescent staining and found the decreased positive cell number when stimulated by LPS. However, it was rescued by ABs in a concentration-dependent manner (Fig. 5c). Meanwhile, we collected supernatant of macrophages and investigated secretory factors like IL-10 and TGF-β by ELISA (Fig. 5d, e). The secretion of both IL-10 and TGF-β was increased by applying ABs. We also investigated the secretion of both IL-10 and TGF-β from ABs alone, and the results showed that the levels of both TGF-β and IL-10 released by ABs are almost undetectable, which demonstrated that macrophages are the source of the molecules (Additional file 3). Next, in the presence of both macrophages and ABs, the expression levels of ARG-1 and TGF-β were increased with concentrations of ABs detecting by western blots (Fig. 5f). Meanwhile, in the presence of both macrophages and ABs, the expression levels of genes like *Il-10*, *Arg-1*, and *Tgf-β* were examined and they all expressed higher when treated by ABs (Fig. 5g–i). Thus, these results indicated that ABs promoted the conversion of macrophages towards the M2 phenotype, which serves as anti-inflammatory macrophages promoting cutaneous wound healing.

### Macrophages treated by ABs further promoted migration and proliferation of fibroblasts

**Fig. 6:**
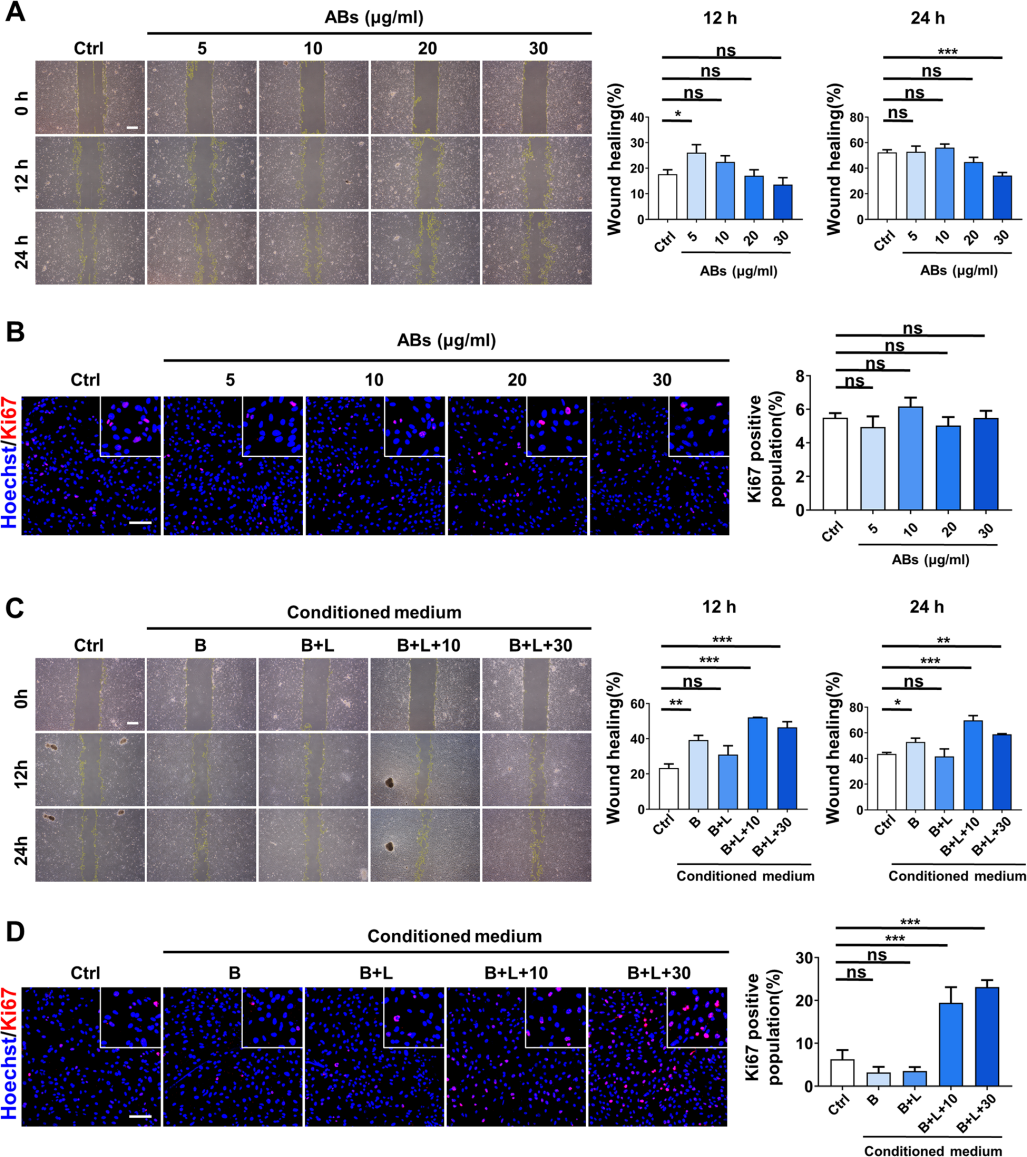
M2 phenotypic macrophages further promoted the functions of fibroblasts. **a** Representative images of scratch assay examining the migration ability of fibroblasts treated with different concentrations of ABs. Scale bar, 500 μm. **b** Representative images of Ki67 expression of fibroblasts treated with different concentrations of ABs. Scale bar, 100 μm. **c** Representative images of scratch assay examining the migration ability of fibroblasts treated with different conditioned media of macrophages. Scale bar, 500 μm. **d** Representative images of Ki67 expression of fibroblasts treated with different conditioned media of macrophages. Scale bar, 100 μm. *n* = 3 per group. Data shown as mean ± SD. *P < 0.05; **P < 0.01; ***P < 0.001; NS, not significant. B, conditioned medium of unstimulated macrophages; B+L, conditioned medium of LPS-stimulated macrophages; B+L+10, conditioned medium of LPS-stimulated macrophages treated with ABs (10 μg/ml); B+L+30, conditioned medium of LPS-stimulated macrophages treated with ABs (30 μg/ml)

Besides macrophages, some other cell types also participate in the cutaneous wound healing process. Fibroblasts are the major component cell types of the dermis and contribute to the new skin tissue formation; we wondered whether ABs affected the function of fibroblasts. We firstly isolated fibroblasts from the skin and treated fibroblasts with different concentrations of ABs. After 24-h treatment, we examined the migration and proliferation abilities by scratch assay and Ki67 immunofluorescence staining, respectively (Fig. 6a, b). However, no significant increase was found in either the migration or the proliferation ability of fibroblasts. In addition to autologous functional transformation, communication between different cell types is also important in tissue repair. Considering the functional conversion of macrophages, we inferred that the converted macrophages further influenced the functions of fibroblasts. Then, we collected the conditioned medium of macrophages and treated fibroblasts with the conditioned medium. Interestingly, the conditioned medium from unstimulated and LPS-stimulated macrophages had no significant effect either on the migration or proliferation abilities of fibroblasts, while the conditioned medium from macrophages treated with ABs enhanced both migration and proliferation abilities of fibroblasts (Fig. 6c, d). To sum up, ABs had no direct effects on fibroblasts whereas macrophages treated with ABs improved both migration and proliferation abilities of fibroblasts, which promoted the cutaneous wound healing process synergistically.

## Discussion

The skin serves as the first defensive line of the human body, so that it is one of the most vulnerable tissues [1]. Therefore, therapies for skin injuries have been highly focused. Among different treatments, stem cell-based therapy shows great potential and is the most popular candidate [2]. Mesenchymal stem cells (MSCs) are the kind of stem cells, which possess the capacity of self-renewal and multi-directional differentiation [24]. MSCs can be isolated from multiple adult mesenchymal tissues like the bone marrow, adipose tissue, and so on [25]. Hence, in contrast with other stem cells, MSCs are much more available and less limited in ethics. Previous studies have demonstrated that exogenous MSCs had therapeutic effects on cutaneous wound healing in different models [26–29]. It has been thought that exogenous MSCs functioned in tissue regeneration through direct differentiation into component cell types of damaged tissue or releasing multiple cytokines and EVs to communicate with other cells via paracrine pathways [7, 25, 30]. Intriguingly, we found that large amounts of exogenous MSCs underwent apoptosis in a short time after transplantation which was consistent with our previous study [9].

Apoptosis is widely known as a manner of programmed cell death, which is not only of great importance in physiological processes like organogenesis and maintaining of tissue homeostasis, but also associated with pathological processes like tumorigenesis and tissue regeneration [11]. In the previous study, we demonstrated that apoptosis enhanced the inflammatory regulatory abilities of infused MSCs partially through activation of caspase-3 [9]. In addition to activation of effector caspase, apoptotic cells present several morphological changes including programmed degradation of DNA, chromatin condensation, cell shrinkage, and plasma blebbing [31]. Apoptotic bodies (ABs) are referred to extracellular vesicles released during apoptosis [32]. As a type of EVs, ABs possess a membrane structure and carry diverse contents including organelles, proteins, mRNAs, and microRNAs [33]. A growing number of studies suggest that extracellular vesicles play an important role in cell-cell communications and functions as the vital mediator of cell signaling in multiple tissue regeneration [34, 35]. In this case, we extracted ABs derived from MSCs and validated the therapeutic effects of ABs in murine cutaneous wound healing models.

A great number of cells undergo apoptosis every day in the human body to ensure cell turnover and sustain tissue homeostasis. To avoid extra damage to the healthy tissue, it is necessary to remove the corpses of apoptotic cells efficiently [15]. There are two categories of phagocytes: non-professional phagocytes like epithelial cells and professional phagocytes like macrophages [12]. Notably, macrophages are the dominant phagocytes that take charge of recognizing and engulfing apoptotic cells or vesicles [14]. Thereby, we asked whether the ABs were mainly phagocytized by macrophages. We labeled the ABs and examined the phagocytosis by macrophages both in vivo and in vitro. As we inferred, ABs could be probably engulfed by macrophages. Classical cutaneous wound healing is a complicated process which includes three overlapping phases: hemostasis and inflammation, newly tissue formation, and tissue remodeling [36]. These phases are highly orchestrated by multiple cell types such as keratinocytes, fibroblasts, immune cells, and endothelial cells. In addition to scavenger cells, macrophages are also immune cells which play significant roles in balancing the inflammatory and repairing processes during the wound healing process [37]. Macrophages are sufficiently flexible cells which polarize to disparate phenotypes in order to adapt to the microenvironment [16]. Accumulating evidence suggests that MSCs and apoptotic cells can trigger the polarization of macrophages towards the M2 phenotype [38–40]. Now that ABs were generated by apoptotic MSCs and largely taken up by macrophages, we then tested the effects of ABs on the phenotypic switch and functional changes of macrophages. The in vitro results showed that ABs could promote polarization of macrophages towards the M2 phenotype. Combined with in vivo results, we inferred that ABs promote cutaneous wound healing at least partially via regulating macrophage polarization.

Since fibroblasts are the major components of the dermis and play important roles in newly tissue repair and tissue remodeling process [41], we wondered if ABs could also affect the functions of fibroblasts. Thus, we investigated the effects of ABs on proliferation and migration abilities of fibroblasts in vitro; however, no significant changes were found. We considered that as non-professional phagocytes, fibroblasts merely possess limited phagocytic abilities [12]. Therefore, unlike macrophages, fibroblasts may not engulf and handle large amounts of ABs. Nevertheless, it has been realized that communication between macrophages and fibroblasts is also a crucial factor in the tissue repair process [37]. Multiple soluble mediators released by macrophages have effects on functions of fibroblasts in the case of tissue repairing [12]. Hence, we further collected the conditioned medium from macrophages and examined the proliferation and migration abilities of fibroblasts. Interestingly, the conditioned medium from macrophages treated with ABs facilitated both proliferation and migration abilities of fibroblasts. We speculate that ABs facilitated the polarization of macrophages towards the M2 phenotype which release cytokines beneficial to the functions of fibroblasts. Taken together, we validated that rather than direct action, ABs can improve the capacity of fibroblasts indirectly through macrophages, which further contribute to the cutaneous wound healing process. In summary, the transplanted MSCs promote cutaneous wound healing partially via ABs which formed during apoptosis. ABs are may largely engulfed by macrophages and thus trigger the polarization of macrophages towards the M2 phenotype. Furthermore, the educated macrophages enhance the functions of fibroblasts, which is also in favor of cutaneous wound healing.

## Conclusions

**Fig. 7:**
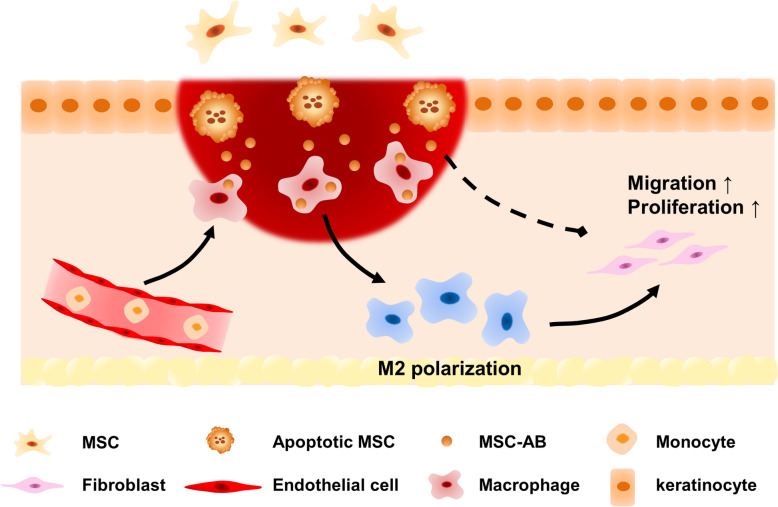
Schema indicating transplanted MSCs undergo apoptosis after transplantation in a mouse skin wound model and releasing apoptotic bodies, converting macrophages towards the M2 phenotype and further enhancing the functions of fibroblasts, together contributing to the cutaneous wound healing process

In this study, we found that ABs accelerated the cutaneous wound healing process, and macrophages were probably the target cells of ABs in vivo. Through in vitro experiments, we proved that ABs promoted macrophage polarization towards the M2 phenotype, and M2 phenotype macrophages further enhanced the functions of fibroblasts, both of which were beneficial for cutaneous wound healing (Fig. 7). Collectively, our findings firstly elucidated that ABs partially play an important role on the therapeutic effects of the administrated MSCs by promoting macrophage polarization. Meanwhile, considering the disadvantages and risks of MSC transplantation such as the low retention rate, safety issues, and the lack of cell sources, we may provide a new strategy for the treatment of cutaneous wound by utilizing ABs which derived from MSCs as a promising candidate to developing cell-free therapy.

## Supplementary Information

**Additional file 1.** Images of histological staining of all the sections from all animals treated by MSCs. (A) Images of the H&E staining of the skin samples. Scale bar, 1 mm in low magnification images, 500 μm in high magnification images. (B) Images of the Masson staining of the skin samples. Scale bar, 1 mm in low magnification images, 500 μm in high magnification images. PBS, phosphate buffer saline; Gel, PF-127 gel; Gel+MSCs, MSCs embedded in PF-127.

**Additional file 2.** Images of histological staining of all the sections from all animals treated by ABs. (A) Images of the H&E staining of the skin samples. Scale bar, 1 mm in low magnification images, 500 μm in high magnification images. (B) Images of the Masson staining of the skin samples. Scale bar, 1 mm in low magnification images, 500 μm in high magnification images. PBS, phosphate buffer saline; Gel, PF-127 gel; Gel+ABs, MSC-ABs embedded in PF-127 gel.

**Additional file 3.** Excluding the source of molecules from ABs. The detection of secretory factors IL-10 and TGF-β released from ABs alone by ELISA.

**Additional file 4.** Additional Tables 1–7 Data tables summarizing the mean and standard deviation of the data obtained.

## Data Availability

Not applicable.

## Abbreviations

MSCs: Mesenchymal stem cells
BMMSCs: Bone marrow mesenchymal stem cells
BMDM: Bone marrow-derived monocyte
EVs: Extracellular vesicles
ABs: Apoptotic bodies
MSC-ABs: MSC-derived ABs
ALP: Alkaline phosphatase
IL-10: Interleukin-10
TGF-β: Transforming growth factor-β
Arg-1: Arginase-1
α-MEM: Alpha-minimum essential medium
DMEM: Dulbecco’s modified Eagle medium
FBS: Fetal bovine serum
BSA: Bovine serum albumin
M-CSF: Macrophage colony-stimulating factor
LPS: Lipopolysaccharide
STS: Staurosporine
PF-127: Pluronic F-127
OCT: Optimal cutting temperature compound
SDS: Sodium dodecyl sulfate-polyacrylamide
PBS: Phosphate-buffered saline
SEM: Scanning electron microscopy
DLS: Dynamic light scattering
TUNEL: TdT-mediated dUTP nick-end labeling
