# Supplementary figures and images for: Apoptotic bodies derived from mesenchymal stem cells promote cutaneous wound healing via regulating the functions of macrophages
Source: Stem Cell Res Ther. 2020 Nov 27;11:507. doi: 10.1186/s13287-020-02014-w (PMC7694913; doi:10.1186/s13287-020-02014-w)

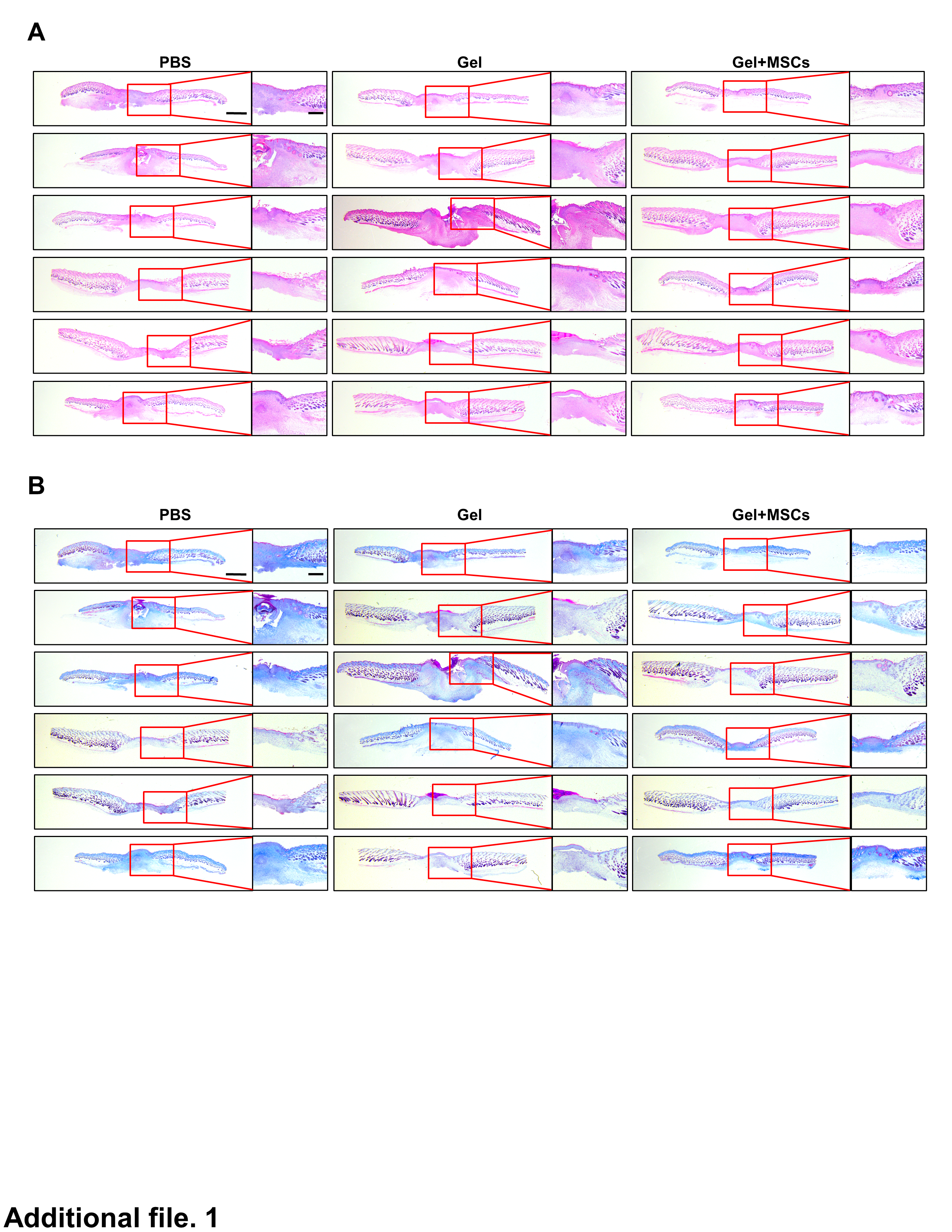

Supplement: Supplementary file 1 — Additional file 1. Images of histological staining of all the sections from all animals treated by MSCs. (A) Images of the H&E staining of the skin samples. Scale bar, 1 mm in low magnification images, 500 μm in high magnification images. (B) Images of the Masson staining of the skin samples. Scale bar, 1 mm in low magnification images, 500 μm in high magnification images. PBS, phosphate buffer saline; Gel, PF-127 gel; Gel+MSCs, MSCs embedded in PF-127. [file 13287_2020_2014_MOESM1_ESM.tif]

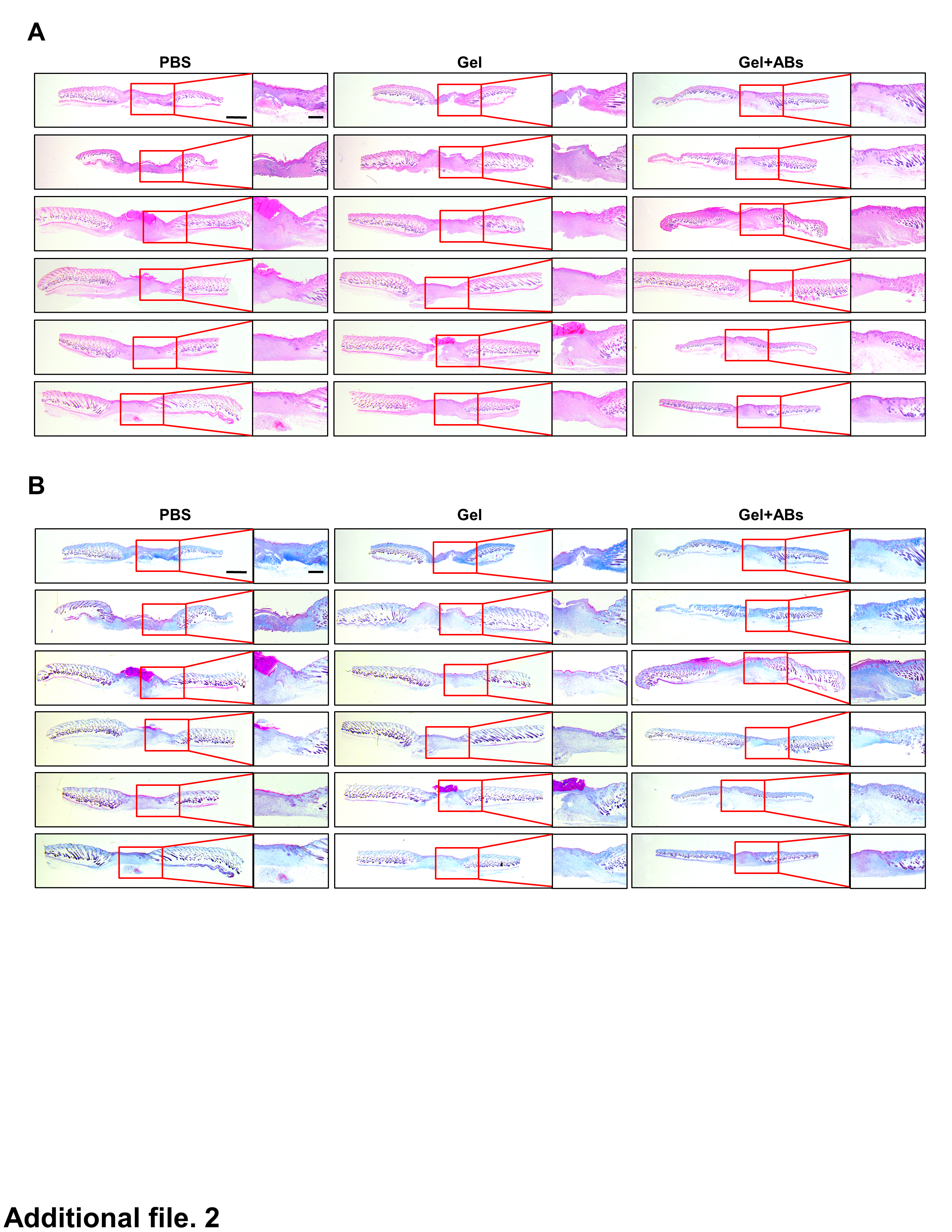

Supplement: Supplementary file 2 — Additional file 2. Images of histological staining of all the sections from all animals treated by ABs. (A) Images of the H&E staining of the skin samples. Scale bar, 1 mm in low magnification images, 500 μm in high magnification images. (B) Images of the Masson staining of the skin samples. Scale bar, 1 mm in low magnification images, 500 μm in high magnification images. PBS, phosphate buffer saline; Gel, PF-127 gel; Gel+ABs, MSC-ABs embedded in PF-127 gel. [file 13287_2020_2014_MOESM2_ESM.tif]

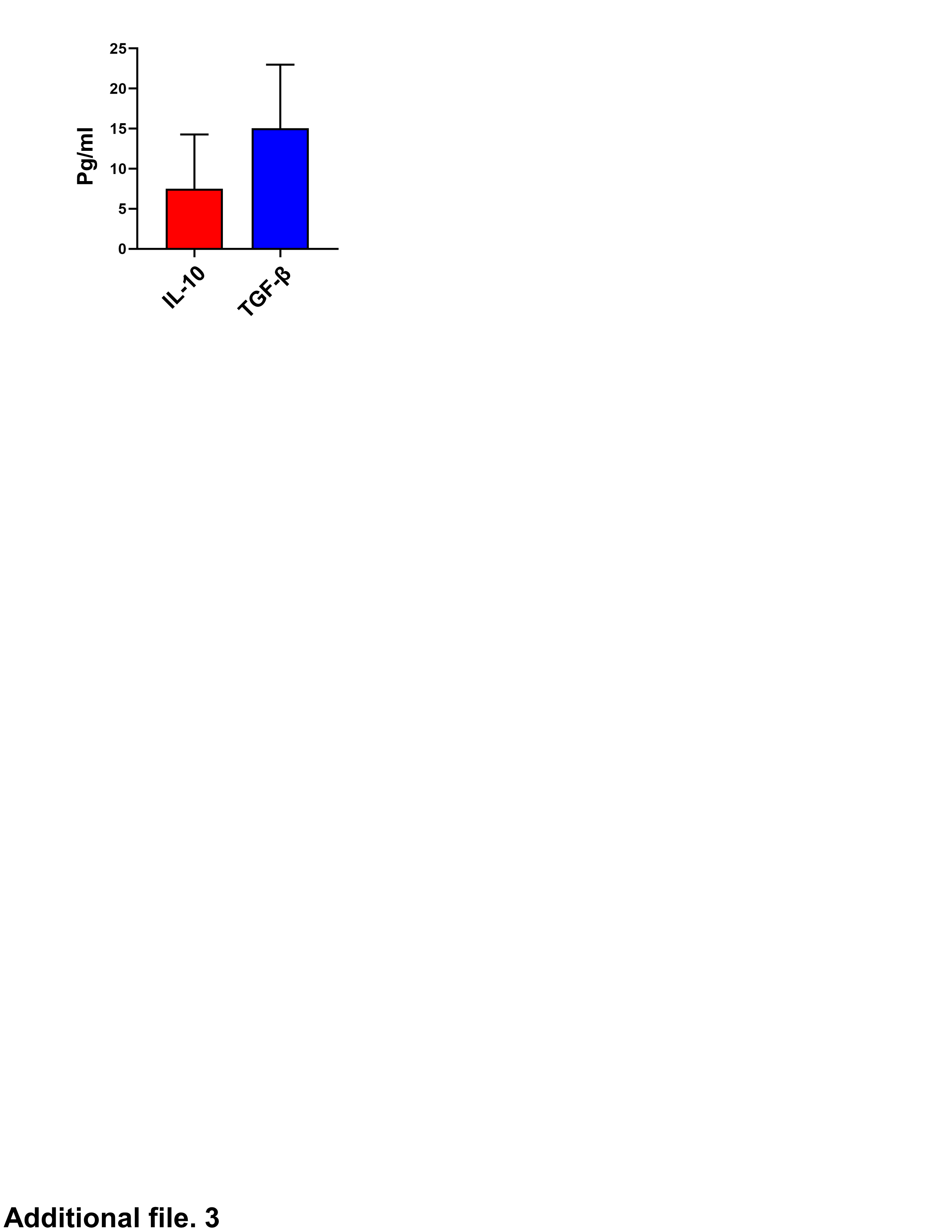

Supplement: Supplementary file 3 — Additional file 3. Excluding the source of molecules from ABs. The detection of secretory factors IL-10 and TGF-β released from ABs alone by ELISA. [file 13287_2020_2014_MOESM3_ESM.tif]

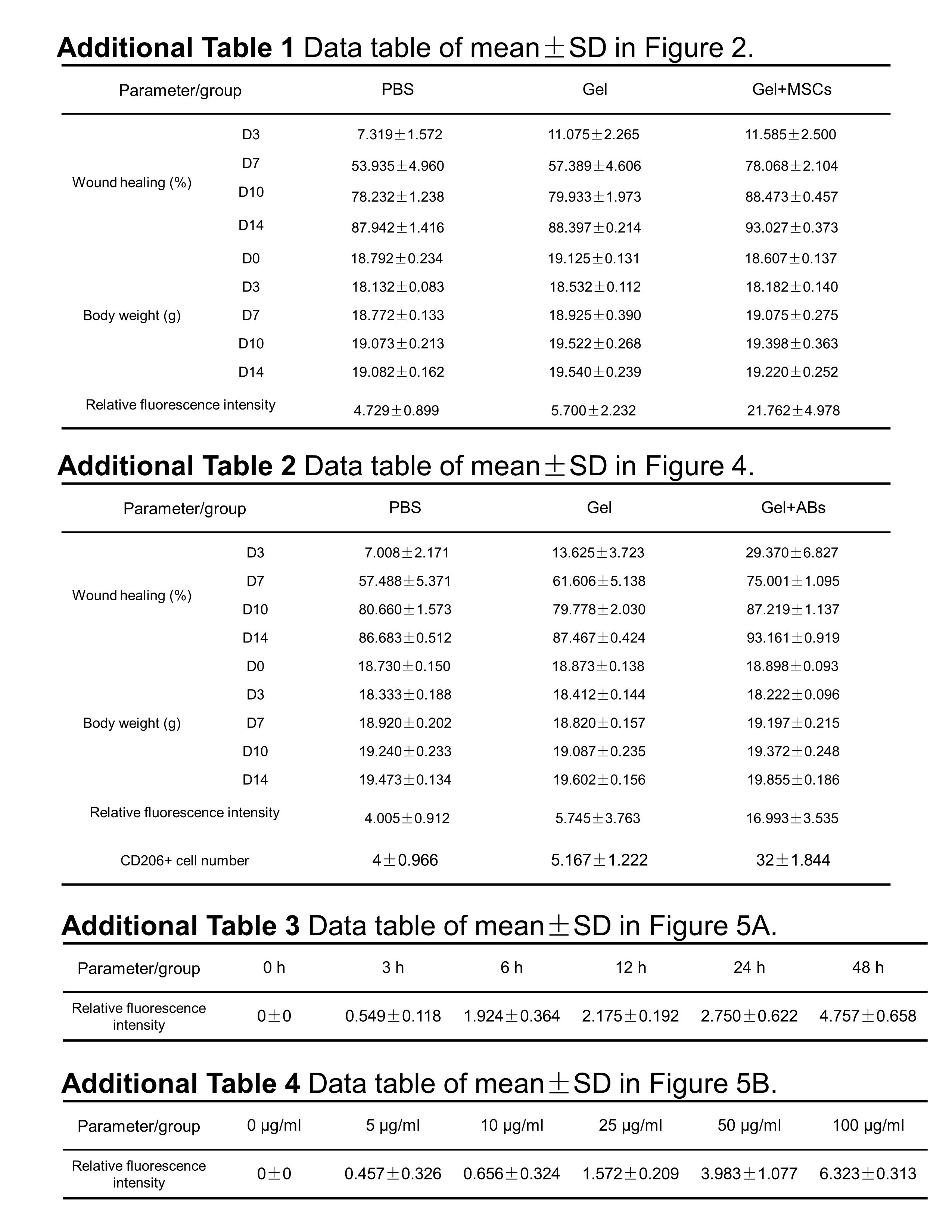

Supplement: Supplementary file 4 — Additional file 4. Additional Tables 1–7 Data tables summarizing the mean and standard deviation of the data obtained. [file 13287_2020_2014_MOESM4_ESM.zip › Additional File 4.TIF]

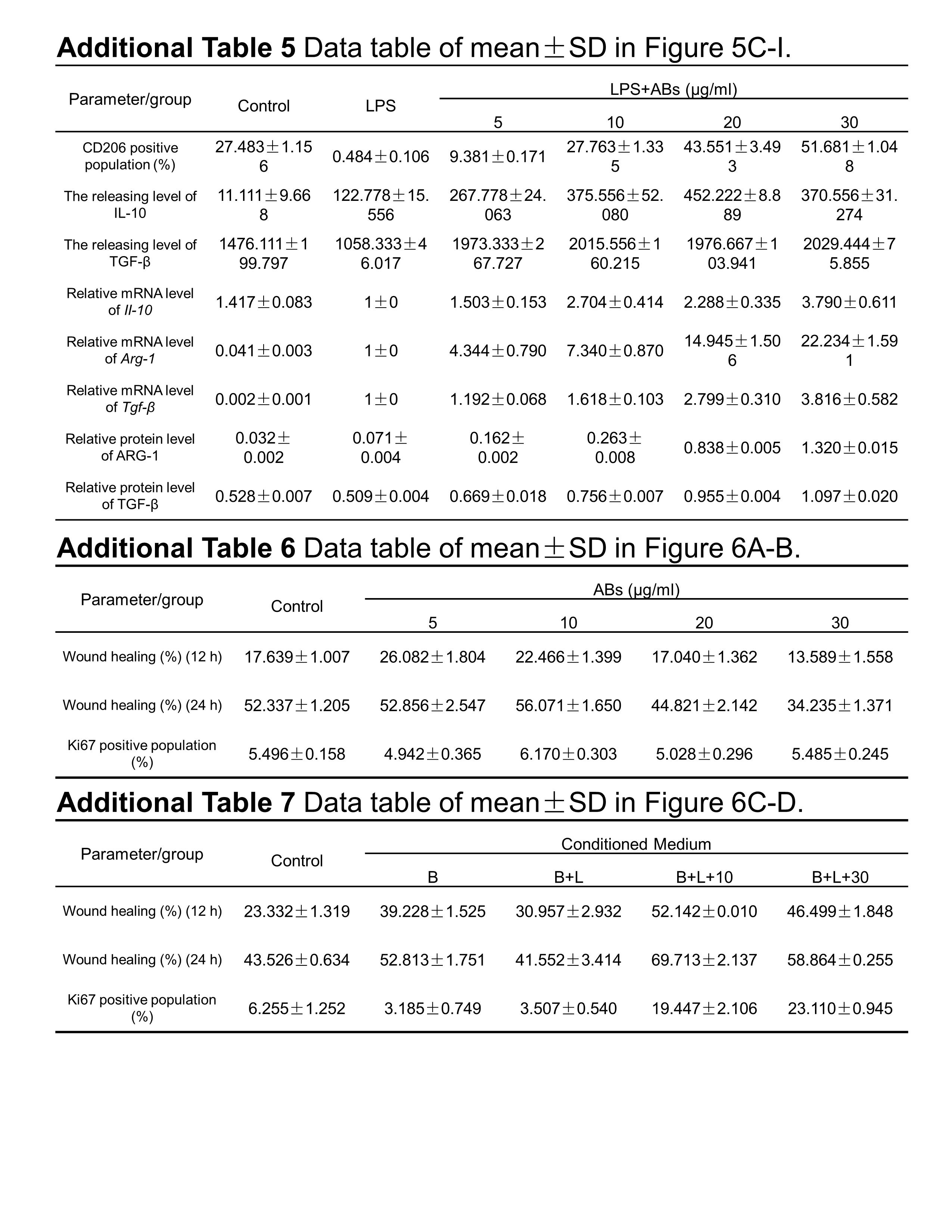

Supplement: Supplementary file 4 — Additional file 4. Additional Tables 1–7 Data tables summarizing the mean and standard deviation of the data obtained. [file 13287_2020_2014_MOESM4_ESM.zip › Additional File 5.TIF]
